# Supplementary material for: Patterns of Intron Gain and Loss in Fungi
Source: PLoS Biol. 2004 Nov 30;2(12):e422. doi: 10.1371/journal.pbio.0020422 (PMC532390; doi:10.1371/journal.pbio.0020422)
Supplement: Table S1 — Also available at http://genes.mit.edu/NielsenEtAl/. (4.3 MB ZIP). [file pbio.0020422.st001.zip › NielsenEtAl/html/1363.html]

AN1766.1.NCU09551.1.MG07444.1.FG05127.1


```
 CLUSTAL W (1.82) Multiple Sequence Alignments - Introns Inserted


Sequence 1: NCU09551.1	601 aa
Sequence 2: MG07444.1	548 aa
Sequence 3: FG05127.1	572 aa
Sequence 4: AN1766.1	636 aa
Alignment Length: 639 aa
Number Identitical Residues: 196 aa
Alignment Score (without introns) 10880


MG07444.1 	--------------~--------------MSSPAAN---ADEHTPLLDGDVPNVPVP---
NCU09551.1	--------------~MTGKITGPGSGNRVLAAPGSSRHVHDETTPLLSESALEANKPTDE
FG05127.1 	--------------~--MRESPSGSAASAMAENGVAP-LSDERTPLLKSNGQGQNPD---
AN1766.1  	MILMLSIPSSTIVA1KFMKKAPSKYQRGCFPGRLLSGEIAFLGRIFLTSPGNVAIVQKRP
          	     : .:::  :      : .      :.              :*          .  

MG07444.1 	-----------~---------------------------------GQQPPPAQSD-----
NCU09551.1	AVRHG------~-----------DGVDIFDDGDTTVVDDDYSTGDGEISVVADDDST---
FG05127.1 	-----------~---------------------------------AEPAIEAQGDDPNQG
AN1766.1  	NFRYWRPYAPM0SFVDPSRPVRCHQLDTLTTILNLTDMSLSQSSPPTQAATARDGSISEQ
          	      . :.  :  ..: .  .   .      .    .  .:.    .  * ... .. 

MG07444.1 	----GDDEVTVVQDEMSKTKLYFILGTSYVGVFLGAVD~GTIFATLSGQISSEFKSLSLM
NCU09551.1	----GDGEITVVADEISTTRLVVIFGTTWVGVFLGAID~ASIIATLSAPIASEFQSLSLL
FG05127.1 	LPAGQEEETTVVAEEVPTAKLILIFGTAWIGVFLGAVD~STIIATLSGPISSEFQSLSLL
AN1766.1  	TPLLAGQSQPPAEEEPSTKRLLLVLCGIWVGVFLAALD1ITIVATLTGPISSSFNSLSLF
          	 .     . . . :* .. :* .::   ::****.*:*  :*.***:. *:*.*:****:

MG07444.1 	SWLATAYLIANAAFQPISGRMTDLFGRGPGLVFSNIMFAAGNLICGLAQTGEIMILGRVV
NCU09551.1	SWIATAYLIANAACQPLSGRMTDIFGRGPGLVFSNVLFSMGNLICGVAKNEQTMILGRVV
FG05127.1 	SWLATAYLISNAACQPISGRLTDIFGRGPGLVFSNLFFAAGNLICGLAHSQTTMIIGRVV
AN1766.1  	SWLATAYLISNAACQPLSGRLTDIFGRKAGLIFSNLVFAAGNLICGLAQRQWTIILGRVI
          	**:******:*** **:***:**:*** .**:***:.*: ******:*:    :*:***:

MG07444.1 	AGIGGGGLMSISTFLGSDLIPLRQRGVFQGIGNVAYG~SGAMLGGVFGGFINDKTALGWR
NCU09551.1	AGIGGGGLMSISTFLGSDLVPLRKRGVVQGLGNIFYG2AGAMLGGVFGGFLNDTSSWGWR
FG05127.1 	AGIGGGGLMSISTFLGSDLVPLRRRGIVQGLGNICYG~SGAMLGGVFGGLINDHTSQGWR
AN1766.1  	AGIGGGGLTAISTFVASDLIPLRKRGVWQGVGNICFG~VGSGLGGVFGGWINDT--LGWR
          	******** :****:.***:***:**: **:**: :*  *: ******* :**    ***

MG07444.1 	LAFLVQVPVVLISAALCFALIRIPPKVSNRSLISRIDFTGVALTISFLVLLLLGLNAGGN
NCU09551.1	LAFLVQVPVSLVSAVLVYFLVKVPPKISNKSLVSRIDFLGSFYIIAFLILLLLGLNAGGN
FG05127.1 	LAFLIQVPPVLVSAVAVHFLVRVPPKQSDKSFLARIDFVGAFLTCSFLVLLLLGLNSGGN
AN1766.1  	WAFLIQIPFVLASAILVALTVKIPPKNANKDKLRRVDFLGAITLVTALVLLLLGLNTGGN
          	 ***:*:*  * **      :::*** :::. : *:** *     : *:*******:***

MG07444.1 	LVPWSHPLVLTTIPLSIVMFLGFIWWESR--TPQPVIPVRLLVDRTILTACFTNLFCSMA
NCU09551.1	VVPWTHPLVLISIPLSLNMLAGFIYWESRPRNTQPVIPVRLLTHRTVLTACLTNFMCTMV
FG05127.1 	IVPWTHPLPLTTIPLAVITFGLFLFWESK--ARQPIIPVKLLLDRTVLNACLCNLACTMT
AN1766.1  	QLPWTHPLILTTLPLSAVVLGAFVYIEAN-IASEPVIPVRLLLNRTVLSGCLTNWFTTMN
          	 :**:*** * ::**:   :  *:: *:.    :*:***:** .**:*..*: *   :* 

MG07444.1 	IFMVIFYIPLYLQVLGYTTADAGLRLIGTPIGTAISSVVGGYAMKKTGRYLGLGISVLLI
NCU09551.1	TLMLTFYIPMYLIVLGHTPTQAGLRLLASPLAICFTSFGSGLVMKYTGKYKMLGIALVST
FG05127.1 	VMGALFYVPLYLQVLGSSATQSGVQILPSPIGISIGSLFSGFVMKKTGKYTRLGVAGLLV
AN1766.1  	VYALLFYLPILFQVQGLSATASGARLIPQAIGTSFGSLGSGALMRYSGRYKMYSHITMLL
          	     **:*: : * * :.: :* :::  .:. .: *. .*  *: :*:*   .   :  

MG07444.1 	KAVGLAVICTLGRDSNSWTPMAGLFLVGMGYGAMLTITMVACVAAVDHSQQAVVTSATY~
NCU09551.1	LVLGVAALTTLGQNSPGWIVFVALFMIGGGYGGMLTVTMVACIAAVDHSQQAVITSATY~
FG05127.1 	LIAGVVVLTVQNQYSPKWLIGVSFFLIGSGYGATLTTTLLACIAAVDHSQQAVITSATY1
AN1766.1  	LVVSSALICTLKPDTPTALPLLYFLLGGVAYGSMLTITLVALISAVDHEHHAVITSASY~
          	   . . : .    :        ::: * .**. ** *::* ::****.::**:***:* 

MG07444.1 	AFRSIGSTLGITVASAIYQNELRRGLWQRFGHLPGAAEEIGRIRDDLGELKRLPDGW~KD
NCU09551.1	AFRSLGATVGITVASAVYQNILKTELWARFGDWPGAAEEIGRIRDDLGELNRLPLAW~RE
FG05127.1 	LARSLGSTVGITIGSAVYQNILKARLWERFGDQPGAADEIHRIRDDLDEINHLPEGC2TR
AN1766.1  	AFRSTGSSIGITIGSSVFQNILRSGLWSRFGDRENAREVISRLLDSLQEIQKVPADW~LP
          	  ** *:::***:.*:::** *:  ** ***.  .* : * *: *.* *::::*      

MG07444.1 	GVIDSFMEGFSGVWYMALSLGLLALVCISLMRQHTLHDTLARK--
NCU09551.1	GVMESFMTAFRGVWFTALGMAAVGLVAVSLMRQHKLHMTLTRQED
FG05127.1 	YTRLLKDGDFHEKDEKTIRYDMDENYTIQHG-NWAISSKDKRHMV
AN1766.1  	GVMDAYMVSLRATFITLLGLAVLGALVSFGMREHVLHSNMARRNE
          	 .       :       :              :  :  .  *:
```
